# Multi-Institutional Retrospective Study of Radiotherapy for Hepatocellular Carcinoma in the Caudate Lobe

**DOI:** 10.3389/fonc.2021.646473

**Published:** 2021-02-26

**Authors:** Sung Uk Lee, Sang Min Yoon, Jason Chia-Hsien Cheng, Tae Hyun Kim, Bo Hyun Kim, Jin-hong Park, Jinhong Jung, Chiao-Ling Tsai, Yun Chiang, Joong-Won Park

**Affiliations:** ^1^Center for Proton Therapy, National Cancer Center, Goyang, South Korea; ^2^Department of Radiation Oncology, Asan Medical Center, University of Ulsan College of Medicine, Seoul, South Korea; ^3^Division of Radiation Oncology, Department of Oncology, National Taiwan University Hospital, National Taiwan University College of Medicine, Taipei, Taiwan; ^4^Center for Liver and Pancreatobiliary Cancer, National Cancer Center, Goyang, South Korea

**Keywords:** hepatocellular carcinoma, caudate lobe, freedom from local progression rate, overall survival, radiotherapy

## Abstract

**Background:** No studies evaluating the clinical outcomes of radiotherapy (RT) for hepatocellular carcinoma (HCC) in the caudate lobe have been available to date. The purpose of this study was to evaluate the effectiveness and safety of RT for HCC in the caudate lobe.

**Material and Methods:** Seventy patients with HCC in the caudate lobe treated with RT from a multi-institutional database were included in this study. The median equivalent dose in 2 Gy (EQD2) was 80.0 Gy_10_ (range, 31.3–99.3), and freedom from local progression (FFLP), progression-free survival (PFS), and overall survival (OS) rates were evaluated.

**Results:** The median time of follow-up was 47.9 months (range, 3.4–127), and the 5-year FFLP, PFS, and OS rates were 80.6% [95% confidence interval (CI), 70.8–91.8], 13.8% (95% CI, 7.5–25.4), and 51.3% (95% CI, 39.9–66.1), respectively. In the multivariate analysis, the radiation dose was significantly associated with the FFLP rate [hazard ratio (HR), 0.57 per 10 Gy_10_ increase, *p* = 0.001], and the status of FFLP was significantly associated with OS (HR, 2.694, *p* = 0.014). The overall rate of ≥grade 3 adverse events was 5.7% (4 of 70), and RT-related mortality was not observed.

**Conclusion:** RT for HCC in the caudate lobe showed promising FFLP and OS rates with safe toxicity profiles. These findings suggest that RT may be a promising treatment option for HCC in the caudate lobe.

## Introduction

Surgical resection is known as the curative treatment for hepatocellular carcinoma (HCC), but it is suitable for <20% of patients for good liver function and performance status (1–3). In particular, the treatment for HCC arising from the caudate lobe is thought to be difficult because of the unique anatomical features of the caudate lobe, such as central and deep locations close to the hepatic hilum and the inferior vena cava and complex vascular supplies and drains (4). With improvements in surgical techniques, surgical resection for HCC in the caudate lobe has been successfully performed, but it is still challenging due to more blood loss, a longer operation time and more complications than HCC in the non-caudate lobe (5–9). Alternatively, percutaneous ablative therapy including radiofrequency ablation (RFA), ethanol injection (PEI), and microwave ablation (MWA) for HCC in the caudate lobe has been tried, but these procedures are also technically difficult because of the deep location and proximity of adjacent major vessels and bile ducts (10–16). In addition, transarterial chemoembolization (TACE) has been one of the treatment options for multiple HCCs, but the complex arterial blood supply of the caudate lobe makes it difficult to achieve local tumor control of HCC in the caudate lobe (17–19). For these reasons, the local tumor control rate of various non-surgical treatment modalities, including RFA, PEI, MWA, and TACE, for HCC in the caudate lobe is lower than that for HCC in the non-caudate lobe (10–20). Thus, to solve the issue of concern regarding the technical difficulty and local tumor control of these local treatment modalities for HCC in the caudate lobe, alternative and complementary treatment options for HCC in the caudate lobe may be needed.

Recently, a better biological understanding of liver tolerance to radiotherapy (RT) and technologic advances in RT techniques, such as three-dimensional conformal RT (3DCRT), intensity-modulated RT (IMRT), stereotactic body RT (SBRT), and proton beam RT (PBT), have made it possible to effectively deliver radiation doses to tumor(s) while sparing surrounding non-cancerous tissues, and its local tumor control effect and safety for HCC patients have been reported (21–30). To the best of our knowledge, no studies have shown the feasibility and effectiveness of RT for HCC in the caudate lobe to date. The incidence of HCC in the caudate lobe is less common than that of HCC in the non-caudate lobe (4, 6, 8), and thus, it is practically difficult to conduct prospective studies to evaluate the feasibility and effectiveness of RT for HCC in the caudate lobe. Based on this background, the purpose of the present study was to evaluate the feasibility and effectiveness of RT for HCC in the caudate lobe using a multi-institutional database.

## Materials and Methods

### Patients

Patients with HCC in the caudate lobe treated with RT at 3 institutes (2 in Korea and 1 in Taiwan) between January 2007 and January 2017 were included in this study. The diagnosis of HCC was based on histologic findings and/or radiologic criteria of the American Association for the Study of Liver Diseases (3), and the location of the tumor was defined based on the Couinaud nomenclature using dynamic computed tomography (CT) and magnetic resonance imaging (MRI) (31). The inclusion criteria of this study were as follows: (i) patients with primary or recurrent/residual HCC lesion(s) in the caudate lobe without vascular invasion who were failed after, were difficult-to-treat with or refused to other treatments; (ii) no prior history of RT to the target lesion(s); (iii) no progressive disease outside of the target lesion(s) at the time of treatment; (iv) no evidence of extrahepatic disease; (v) Child-Pugh score ≤ 7 without uncontrolled ascites; and (vi) Eastern Cooperative Oncology Group (ECOG) performance status (PS) of 0–2. The data of each patient, including age, sex, ECOG PS, tumor size, pre-treatment serum α-fetoprotein (AFP) concentration, RT technique, total radiation dose [equivalent doses in a 2 Gy fraction (EQD2) calculated using a linear quadratic model with a α/β ratio of 10 or 3 for acute and late effects on tumor and organs at risk, respectively (32)], pre- and post-treatment modalities, sites and time of disease progression, and follow-up data were collected from medical records. The collected data were managed by assigning case numbers to each participating institute and anonymizing them. Data analysis was performed centrally, and all methods were performed in accordance with the relevant regulations and guidelines. This study was approved by the institutional review board of each participating institute and complied with the Declaration of Helsinki and Good Clinical Practice guidelines. The requirement for written informed consent was waived due to the retrospective study design.

### Assessments and Statistical Analysis

The equations should be inserted in editable format from the equation editor. Local progression was defined as the presence of regrowth or a new tumor within the planning target volume (PTV) to the target lesion(s) in the caudate lobe. Intrahepatic progression was defined as the presence of regrowth of previously treated non-target lesion(s) or a new intrahepatic tumor within the liver outside of the PTV, and extrahepatic progression was defined as regrowth or a new tumor at extrahepatic sites, including regional and non-regional lymph nodes and distant organs. The adverse events (AEs) related to RT were assessed according to the Common Terminology Criteria for Adverse Events version 4.03 (https://ctep.cancer.gov/protocolDevelopment/electronic_applications/docs/CTCAE_4.03.xlsx). The times of freedom from local progression (FFLP), progression-free survival (PFS) and overall survival (OS) were determined from the commencement date of RT to the date of local progression, any disease progression or death, and death from any cause or the last follow-up, respectively. The distributions of continuous and categorical variables were compared with the *t*-test and Fisher's exact test, respectively, and correlations among variables were assessed using Spearman's rank correlation coefficient test. The probability of survival was estimated using the Kaplan-Meier method, and the differences in survival were compared using the log-rank test in univariate analysis. In multivariate analysis using a stepwise forward selection procedure with variables with *p* < 0.1 in the univariate analysis, the hazard ratios (HRs) were estimated using a Cox proportional hazards model. A *p*-value < 0.05 was considered statistically significant, and all statistical analyses were performed with STATA software (version 14.0; StataCorp, College Station, TX).

## Results

A total of 70 patients who met the inclusion criteria from the multi-institutional database were identified and included in this study (Table 1). There were 56 (80%) males and 14 (20%) females. The median age was 62 years (range, 31–88), and the median size of the tumor in the caudate lobe was 2.0 cm (range, 1.0–8.2). At the time of RT for HCC in the caudate lobe, most (*n* = 67, 95.7%) patients, except for 3 (4.3%) who were treatment naïve (by technical infeasibility) or refused other local treatments, had a recurrent and/or residual tumor in the caudate lobe, and the median interval from the initial diagnosis of HCC to the detection of an HCC lesion in the caudate lobe was 26.3 months (range, 0.4–198.1). Pre-treatments for the target and non-target lesion(s) are summarized in Table 1. Of the 70 patients, 13 (18.6%) received 3DCRT, 11 (15.7%) received IMRT, 21 (30.0%) received SBRT, and 25 (35.7%) received PBT. The median radiation dose delivered to the target lesion was 51.8 Gy (range, 30.0–70.0), with a median fraction size of 6.0 Gy (range, 2.0–15). Because of heterogeneity of the dose-fractionation regimens, we used EQD2 for analysis, and the median EQD2 was 80.00 Gy_10_ (range, 31.3–99.3). The median EQD2 for the treatment methods was 54.0 Gy_10_ (range, 31.3–62.5) for 3DCRT, 57.3 Gy_10_ (range, 47.3–71.5) for IMRT, 93.8 Gy_10_ (range, 53.6–93.8) for SBRT and 91.3 Gy_10_ (range, 62.5–99.2) for PBT (*p* < 0.001).

**Table 1:** Patient and treatment characteristics.

**Characteristic**		**Distribution, *n* (%)**
Sex	Male	56 (80)
	Female	14 (20)
Age, years	Median (range)	62 (31–88)
	≤ 60	32 (45.7)
	>60	38 (54.3)
ECOG PS	0	56 (80)
	1	14 (20)
Child-Pugh	A	63 (90)
classification	B7	7 (10)
Etiology	HBV	44 (62.9)
	Others	26 (37.1)
Tumor size, cm	Median (range)	2.0 (0.8–8.2)
	<2	31 (44)
	2–3.9	25 (36)
	≥4	14 (20)
AFP, ng/mL	Median (range)	8.0 (1.0–80,000)
	≤ 200	54 (77.1)
	>200	16 (22.9)
Diagnosis	Pathologic	28 (40)
	Image based	42 (60)
Pre-Tx to target lesion(s)	No	21 (30)
	Yes	49 (70)
	TACE	45 (64.3)
	TACE + RFA/PEI	4 (5.7)
Pre-Tx to non-target lesion(s)	No	11 (15.7)
	Yes	59 (84.3)
	SR ± TACE/RFA/PEI	28 (40.0)
	TACE ± RFA/PEI	25 (35.7)
	RFA	5 (7.1)
	SAT^*^± TACE	1 (1.4)
RT technique	3DCRT	13 (18.6)
	IMRT	11 (15.7)
	SBRT	21 (30.0)
	PBT	25 (35.7)
Radiation dose, Gy_10_^†^	Median (range)	80 (21.3–99.2)
Planning target volume, cm^3^^§^	Median (range)	32.3 (10.0–669.9)
Total liver (TL) volume, cm^3^^§^	Median (range)	1238.6 (529.1–1943.1)
_TL_V30Gy, %	Median (range)	8.5 (1.5–45.2)
Remaining liver (RL)^‡^ volume, cm^3^^§^	Median (range)	1214.5 (526.3–1903.4)
_RL_V30Gy, %	Median (range)	7.3 (1.3–30.6)
_RL_AV30Gy, cm^3^	Median (range)	1098.2 (484.7–1772.4)
Gastrointestinal (GI) organs^§^		
_GI_V45Gy, cm^3^	Median (range)	0.0 (0.0–4.3)
_GI_V50Gy, cm^3^	Median (range)	0.0 (0.0–1.3)
_GI_Dmax, Gy_10_	Median (range)	29.8 (56.2–61.0)

*n, number of patients; ECOG PS, eastern cooperative oncology group performance status; HBV, hepatitis B virus; AFP, α-fetoprotein; Tx, treatment; TACE, transarterial chemoembolization; RFA, radiofrequency ablation; PEI; percutaneous ethanol injection; SR, surgical resection; SAT, systemic anticancer therapy; RT, radiotherapy; 3DCRT, 3-dimensional conformal RT; IMRT, intensity-modulated RT; SBRT, stereotactic body RT; PBT, proton beam RT; _TL_V30Gy, relative volume of the total liver receiving ≥30 Gy_3_; _RL_V30Gy, relative volume of the remaining liver receiving ≥30 Gy_3_; _RL_AV30Gy, absolute volume of the remaining liver receiving <30 Gy_3_; _GI_V45Gy and _GI_V50Gy, absolute volume of gastrointestinal (GI) organs receiving ≥45 Gy_10_ and ≥50 Gy_10_, respectively; _GI_Dmax, delivered radiation dose to GI organs*.

* *Sorafenib (n = 1)*.

† *Equivalent doses in the 2 Gy fraction calculated using a linear quadratic model with a α/β ratio of 10 or 3 for acute and late effects on tumor and organs at risk, respectively (32)*.

‡ *Remaining liver = total liver – tumor*.

§ *Dose-volumetric parameters were available in 68 patients*.

The median time of follow-up was 47.9 months (range, 3.4–127), and at the time of analysis, 37 patients were alive, and 33 had died from disease progression. Of 70 patients, disease progression occurred in 58 (82.6%), and the patterns of disease progression were as follows (Supplementary Figure 1): the initial sites of disease progression were local sites in 5 (7.1%) patients, intrahepatic sites in 48 (67.1%), and extrahepatic sites in 11 (15.7%); the all sites of disease progression were local sites in 11 (15.7%) patients, intrahepatic sites in 53 (75.7%), and extrahepatic sites in 21 (29.9%). Of 53 patients who experienced disease progression, the median times to local, intrahepatic, and extrahepatic progression were 13.6 months (range, 4.0–39.2), 8.5 months (range, 0.3–64.8), and 13.7 months (range, 0.4–79.3), respectively. After disease progression occurred, subsequent treatment, such as local and/or systemic therapy, either alone or in combination, was performed (Supplementary Table 1). The median time of FFLP was not yet reached, and those of PFS and OS were 9.5 months [95% confidence interval (CI), 4.9–14.0] and 80.2 months (95% CI, 52.4–107.9), respectively. The actuarial 1-, 3-, and 5-year FFLP rates were 93.9% (95% CI, 88.0–99.8), 85.0% (95% CI, 75.8–94.2), and 80.6% (95% CI, 70.8–91.8), respectively. The actuarial 1-, 3-, and 5-year PFS rates were 45.4% (95% CI, 33.6–57.2), 22.0% (95% CI, 12.2–31.8), and 13.8% (95% CI, 7.5–25.4), respectively. The actuarial 1-, 3-, and 5-year OS rates were 85.6% (95% CI, 77.4–93.8), 68.8% (95% CI, 57.6–80.0), and 51.3% (95% CI, 39.9–66.1), respectively (Supplementary Figure 2).

The FFLP rates significantly increased with increasing radiation dose, and the differences in FFLP rates were most significant when the cutoff value of the radiation dose was 90 Gy_10_ (Table 2). The patients with FFLP had significantly longer median OS times (82.8 months vs. 35.6 months) and 5-year OS rates (59.4 vs. 13.6%) than the patients without FFLP (*p* = 0.033) (Table 3). Similarly, PFS showed a longer trend in patients with FFLP than in those without FFLP, but the difference was not significant (*p* > 0.05) (Table 3). In the univariate analysis, tumor size, a history of treatment to non-target lesion(s), and the radiation dose were significantly related to FFLP; the serum level of AFP and a history of treatment to non-target lesion(s) were significantly related to PFS; and the Child-Pugh classification, serum level of AFP, radiation dose, and status of FFLP were significantly related to OS (*p* < 0.05 each) (Table 3). The results of the multivariate analysis were as follows: the radiation dose was a significant factor for FFLP; the serum level of AFP was a significant factor for PFS; and the Child-Pugh classification, serum level of AFP, and status of FFLP were significant factors for OS (*p* < 0.05 each) (Table 4).

**Table 2:** Comparison of the freedom from local progression (FFLP) rate according to the radiation dose.

	**FFLP rate**		
Cutoff value^*^ **of the radiation dose, Gy**_**10**_	***n***	**5-year (95% CI)**	***p*****-value**^**†**^
≤ 50	9	55.6 (27.4–100.0)	0.051
>50	61	83.9 (74.2–94.9)	
≤ 60	19	55.9 (34.6–90.5)	0.009
>60	51	88.4 (79.2–98.7)	
≤ 70	25	63.4 (44.9–89.6)	0.021
>70	45	89.1 (79.3–100)	
≤ 90	40	65.7 (50.9–84.9)	0.002
>90	30	100.0 (-)	

*CI, confidence interval; n, number of patients; ECOG PS, eastern cooperative oncology group performance status; HBV, hepatitis B virus; AFP, α-fetoprotein; Tx, treatment; TACE, transarterial chemoembolization; RFA, radiofrequency ablation; PEI; percutaneous ethanol injection; SR, surgical resection; SAT, systemic anticancer therapy; RT, radiotherapy; 3DCRT, 3-dimensional conformal RT; IMRT, intensity-modulated RT; SBRT, stereotactic body RT; PBT, proton beam RT; _TL_V30Gy, relative volume of the total liver receiving ≥30 Gy_3_; _RL_V30Gy, relative volume of the remaining liver receiving ≥30 Gy_3_; _RL_AV30Gy, absolute volume of the remaining liver receiving <30 Gy_3_; _GI_V45Gy and _GI_V50Gy, absolute volume of gastrointestinal (GI) organs receiving ≥45 Gy_10_ and ≥50 Gy_10_, respectively; _GI_Dmax, delivered radiation dose to GI organs*.

* *No patient received a radiation dose of 80–90 Gy_10_*.

† *log-rank test*.

**Table 3:** Univariate analysis of pre-treatment characteristics and treatment factors for the freedom from local progression (FFLP), progression-free survival (PFS), and overall survival (OS) rates.

			**FFLP rate**		**PFS rate**		**OS rate**	
**Characteristic**		***n***	**5-year (95% CI), %**	***p***-value^*^	**5-year (95% CI), %**	***p***-value^*^	**5-year (95% CI), %**	***p***-value^*^
Sex	Male	56	83.0 (72.8–94.6)	0.428	15.3 (8.1–28.8)	0.737	55.4 (42.6–72.0)	0.489
	Female	14	69.4 (44.8–100)		7.9 (1.2–51.9)		35.2 (16.2–76.1)	
Age	≤ 60	32	69.7 (54.0–90.0)	0.065	17.9 (8.3–38.2)	0.524	52.4 (36.8–74.8)	0.706
	>60	38	91.0 (81.7–100)		10.3 (3.8–27.7)		49.9 (34.6–72.0)	
Child-Pugh classification	A	63	80.8 (70.7–92.4)	0.709	13.7 (7.2–26.2)	0.996	55.5 (43.3–71.2)	0.003
	B	7	85.7 (63.3–100)		14.3 (2.3–87.7)		14.3 (2.3–87.7)	
Etiology	HBV	44	76.1 (63.4–91.3)	0.286	8.2 (2.9–23.0)	0.337	54.8 (40.3–74.6)	0.128
	Others	26	90.2 (78.2–100)		23.8 (11.6–48.9)		44.5 (27.9–70.8)	
Tumor size, cm	<2	31	95.5 (87.1–100)	0.014	12.1 (4.5–32.3)	0.644	64.5 (48.7–85.4)	0.195
	2-3.9	25	64.2 (46.9–87.9)		10.7 (3.2–35.5)		39.4 (23.5–66.1)	
	≥4	14	76.2 (52.1–100)		25.0 (9.6–65.1)		47.9 (25.4–90.3)	
AFP, ng/mL	≤ 200	54	81.9 (71.2–94.3)	0.365	15.6 (8.2–29.6)	0.022	56.0 (42.8–73.3)	0.027
	>200	16	77.0 (56.8–100)		7.5 (1.2–48.4)		33.7 (16.5–68.6)	
Pre-Tx to target lesion(s)	No	21	87.7 (73.0–100)	0.352	20.4 (8.5–48.8)	0.141	63.8 (45.4–89.7)	0.318
	Yes	49	77.8 (65.7–92.0)		11.5 (5.2–25.5)		46.1 (32.8–64.7)	
Pre-Tx to non-target lesion(s)	No	11	58.9 (34.6–100)	0.035	30.7 (12–78.3)	0.032	50.5 (27.3–93.3)	0.882
	Yes	59	84.7 (74.8–96.0)		10.9 (5.1–23.4)		51.5 (39.0–68.0)	
Radiation dose, Gy_10_	≤ 90	40	65.7 (50.9–84.9)	0.002	12.5 (5.5–28.4)	0.535	42.9 (29.6–62.1)	0.044
	>90	30	100.0 (-)		14.7 (5.6–38.5)		65.1 (47.6–88.9)	
Status of FFLP	Yes	59	-	-	16.5 (9.1–30.0)	0.055	59.4 (47.0–75.0)	0.033
	No	11	-		[n] •- )		13.6 (2.5–73.8)	

*n, number of patients; ECOG PS, eastern cooperative oncology group performance status; HBV, hepatitis B virus; AFP, α-fetoprotein; Tx, treatment; TACE, transarterial chemoembolization; RFA, radiofrequency ablation; PEI; percutaneous ethanol injection; SR, surgical resection; SAT, systemic anticancer therapy; RT, radiotherapy; 3DCRT, 3-dimensional conformal RT; IMRT, intensity-modulated RT; SBRT, stereotactic body RT; PBT, proton beam RT; _TL_V30Gy, relative volume of the total liver receiving ≥30 Gy_3_; _RL_V30Gy, relative volume of the remaining liver receiving ≥30 Gy_3_; _RL_AV30Gy, absolute volume of the remaining liver receiving <30 Gy_3_; _GI_V45Gy and _GI_V50Gy, absolute volume of gastrointestinal (GI) organs receiving ≥45 Gy_10_ and ≥50 Gy_10_, respectively; _GI_Dmax, delivered radiation dose to GI organs. CI, confidence interval*.

* *log-rank test*.

**Table 4:** Multivariate analysis of factors influencing the freedom from local progression (FFLP), progression-free survival (PFS), and overall survival (OS) rates.

		**Hazard ratio**	**95% CI**	*p*-value^*^
**FFLP rate**				
Radiation dose, Gy_10_	Per 10 Gy_10_ increase	0.638	0.475–0.856	0.003
**PFS rate**				
AFP, ng/mL	≤ 200	1.000		0.043
	>200	1.865	1.019–3.414	
**OS rate**				
Child-Pugh classification	A	1.000	-	0.001
	B	5.391	2.082–13.956	
AFP, ng/mL	≤ 200	1.000		0.009
	>200	2.740	1.293–5.807	
Status of FFLP	Yes	1.000		0.014
	No	2.694	1.220–5.947	

*n, number of patients; ECOG PS, eastern cooperative oncology group performance status; HBV, hepatitis B virus; AFP, α-fetoprotein; Tx, treatment; TACE, transarterial chemoembolization; RFA, radiofrequency ablation; PEI; percutaneous ethanol injection; SR, surgical resection; SAT, systemic anticancer therapy; RT, radiotherapy; 3DCRT, 3-dimensional conformal RT; IMRT, intensity-modulated RT; SBRT, stereotactic body RT; PBT, proton beam RT; _TL_V30Gy, relative volume of the total liver receiving ≥30 Gy_3_; _RL_V30Gy, relative volume of the remaining liver receiving ≥30 Gy_3_; _RL_AV30Gy, absolute volume of the remaining liver receiving <30 Gy_3_; _GI_V45Gy and _GI_V50Gy, absolute volume of gastrointestinal (GI) organs receiving ≥45 Gy_10_ and ≥50 Gy_10_, respectively; _GI_Dmax, delivered radiation dose to GI organs. CI, confidence interval*.

* *Multivariate analysis using the Cox proportional hazards model*.

After the completion of RT, hepatic AEs were observed in 23 (32.9%) patients: grade 1 in 17 (24.3%) patients, grade 2 in 5 (7.1%), and grade 3 in 1 (1.4%), and gastrointestinal (GI) AEs were observed in 12 (17.1%) patients: grade 1 in 4 (5.7%) patients, grade 2 in 5 (7.1%), grade 3 in 2 (2.9%), and grade 4 in 1 (1.4%). The overall rate of ≥grade 3 AEs was 5.7% (4 of 70), and RT-related mortality was not observed. One patient with grade 3 hepatic AEs showed asymptomatic liver function abnormality, and it became normalized spontaneously within 1 month. Among 3 patients with ≥Grade 3 GI AEs, one patient showed grade 3 nausea which require hospitalization for supportive care, and two patients experienced upper GI bleeding which received endoscopic argon laser coagulation with transfusion (grade 3) and received surgical intervention (grade 4), respectively. The other patients who had grade 1-2 hepatic and GI AEs were self-limited and easily managed by supportive care. Overall, median intervals from the completion date of RT to observed date of AEs were 2.1 (0.1–4.1) months and 1.2 (0.1–4.8) months regarding hepatic AEs and GI AEs, respectively. Of 70 patients, dose volumetric parameters for the liver and GI organs were available in 68 (97.1%). The incidence of ≥grade 2 hepatic AEs significantly increased with increasing relative volumes of the total liver and remaining liver receiving ≥30 Gy_10_ (_TL_V30Gy and _RL_V30Gy, respectively) (*p* < 0.05 each), and the incidence of ≥grade 2 GI AEs increased with an increasing PTV, absolute volumes of GI organs receiving ≥45 Gy_10_ (_GI_V45Gy), and maximum doses to GI organs (_GI_Dmax) (*p* < 0.05 each) (Table 5). The radiation dose was negatively correlated with the PTV (*r* = −0.574, *p* < 0.001), and the PTV, _TL_V30Gy, _RL_V30Gy, _GI_V45Gy, and _GI_Dmax were correlated with each other (PTV vs. _TL_V30Gy: *r* = 0.688, *p* < 0.001; PTV vs. _RL_V30Gy: *r* = 0.610, *p* < 0.001; PTV vs. _GI_V45Gy: *r* = 0.488, *p* < 0.001; PTV vs. _GI_Dmax: *r* = 0.611, *p* < 0.001; _TL_V30Gy vs. _RL_V30Gy: *r* = 0.949, *p* < 0.001; _GI_V45Gy vs. _GI_Dmax: *r* = 0.753, *p* < 0.001).

**Table 5:** Incidence of ≥grade 2 hepatic and gastrointestinal (GI) adverse events (AEs) according to dose-volumetric parameters (*n* = 68).

		** < Grade 2 hepatic AEs, n (%)**	**≥Grade 2 hepatic AEs, n (%)**	***p*-value**
Radiation dose, Gy_10_	μ ±σ	76.3 ± 18.1	59.8 ± 19.2	0.038^*^
Planning target volume, cm^3^	μ ±σ	71.9 ±116.2	220.5 ± 230.6	0.225^*^
Total liver (TL) volume, cm^3^	μ ±σ	1243.1 ± 327.0	1265.0 ± 261.1	0.884^*^
_TL_V30Gy, %	μ ±σ	10.1 ± 7.9	18.7 ± 4.8	0.020^*^
	≤ 15	53 (98.1)	1 (1.9)	0.005^†^
	>15	10 (71.4)	4 (28.6)	
Remaining liver (RL)^‡^ volume, cm^3^	μ ±σ	1208.9 ± 317.1	1200.6 ± 246.3	0.954^*^
_RL_V30Gy, %	μ ±σ	8.5 ± 6.6	14.2 ± 3.4	0.061^*^
	≤ 10	52 (98.1)	1 (1.9)	0.007^†^
	>10	11 (73.3)	4 (26.7)	
_RL_AV30Gy, cm^3^	μ ±σ	1106.2 ± 301.5	1033.4 ± 239.8	0.601^*^
		** < Grade 2 GI AEs, n (%)**	**≥Grade 2 GI AEs, n (%)**	***p*****-value**
Radiation dose, Gy_10_	μ ±σ	77.4 ± 18.1	55.7 ± 10.7	<0.001^*^
Planning target volume, cm^3^	μ ±σ	60.3 ± 101.9	316.5 ± 179.2	0.017^*^
_GI_V45Gy, cm^3^	μ ±σ	0.7 ± 2.9	1.5 ±1.7	0.498^*^
	≤ 1.0	56 (94.9)	3 (5.1)	0.027^†^
	>1.0	6 (66.7)	3 (33.3)	
_GI_V50Gy, cm^3^	μ ±σ	0.3 ± 1.8	0.3 ± 0.5	0.911^*^
_GI_Dmax, Gy_10_	μ ±σ	28.8 ± 15.2	48.7 ± 6.9	<0.001^*^
	≤ 45.0	50 (92.2)	2 (3.8)	0.024^†^
	>45.0	12 (75.0)	4 (25.0)	

*μ, mean; σ, standard deviation; n, number of patients; ECOG PS, eastern cooperative oncology group performance status; HBV, hepatitis B virus; AFP, α-fetoprotein; Tx, treatment; TACE, transarterial chemoembolization; RFA, radiofrequency ablation; PEI; percutaneous ethanol injection; SR, surgical resection; SAT, systemic anticancer therapy; RT, radiotherapy; 3DCRT, 3-dimensional conformal RT; IMRT, intensity-modulated RT; SBRT, stereotactic body RT; PBT, proton beam RT; _TL_V30Gy, relative volume of the total liver receiving ≥30 Gy_3_; _RL_V30Gy, relative volume of the remaining liver receiving ≥30 Gy_3_; _RL_AV30Gy, absolute volume of the remaining liver receiving <30 Gy_3_; _GI_V45Gy and _GI_V50Gy, absolute volume of gastrointestinal (GI) organs receiving ≥45 Gy_10_ and ≥50 Gy_10_, respectively; _GI_Dmax, delivered radiation dose to GI organs. CI, confidence interval*.

* *t-test*.

† *Fisher's exact test*.

## Discussion

Surgical resection of the caudate lobe with adjacent hemilobes or other segments to achieve complete resection with sufficient margins may be feasible in HCC patients with good liver function, but limited surgical resection of the caudate lobe has been attempted because most HCC patients have chronic hepatitis or cirrhosis (5–9). Previous studies on surgical resection for HCC in the caudate lobe have shown that surgical resection might be feasible and potentially curative, with 5-year OS rates of 25.9–76% (5–9). However, surgical resection is still more technically difficult in the caudate lobe than in the non-caudate lobe and is frequently impossible to perform in HCC patients due to concerns about residual liver function after resection. Thus, nonsurgical treatment modalities, such as ablative treatments (i.e., RFA, PEI, and MWA) and TACE, either alone or in combination, have been attempted, and the studies regarding non-surgical treatments for HCC in the caudate lobe are summarized in Table 6 (10–20). Due to the complicated anatomical features of the caudate lobe, such as proximity to major vessels and bile ducts and its deep location, introducing an electrode or needle into tumor(s) during ablative procedures may be technically difficult or, in some cases, impossible to perform. In particular, in RFA, local ablative ability is potentially impaired by the heat sink effect resulting from adjacent major vessels. Thus, ablative treatments for HCC in the caudate lobe, either alone or in combination, have shown 3-year local progression rates of 2.4–37.3% and 3- and 5-year OS rates of 43.7–86.6% and 11.2–80.8%, respectively, which were inferior to those for HCC in the non-caudate lobe (10–14, 16). Alternatively, TACE has been attempted for HCC in the caudate lobe, but obtaining a sufficient therapeutic effect of TACE is more difficult in the caudate lobe than in the non-caudate lobe due to the complexity of arterial supplies to HCC in the caudate lobe. TACE for HCC in the caudate lobe has shown 3-year local progression rates of 38.5–75% and 3-year OS rates of 62–73% (18, 19). To mutually complement each limitation of ablative treatments and TACE, combinations of ablative treatment and TACE have also been tried, with 3-year local progression rates of 13.5–28% and 3-year OS rates of 48.1–80.8% (15, 17, 20). The technical feasibility and success rate of ablative treatments and TACE may be influenced by the complicated anatomical features of the caudate lobe, and local tumor control is still one of the remaining concerns. Thus, an effective local treatment option for HCC in the caudate lobe that is less influenced by the unique anatomical features of the caudate lobe is needed.

**Table 6:** Studies on non-surgical treatments for HCC in the caudate lobe.

**References**				**Tumor size, cm**	**LP rate, %**				**OS rate, %**				
	**Modality**	***n***	**CPC, %**	**Median (range)**	**1-y**	**2-y**	**3-y**	**5-y**	**1-y**	**2-y**	**3-y**	**5-y**	**Adverse events (AEs)**
Liu et al. (13)	RFA	33	A, 91.8	2.6 ± 1.1^§^ (1.0–5.0)	17.3	-	37.3	37.3	79.4		43.7	11.2	5.5% major AEs
	PEI	23	B, 8.2										
	RFA+PEI	17											
Nishigaki et al. (14)	RFA	20	-	1.7 ± 0.5^§^ ( ≤ 3)	-	22.3	22.3	-	-	-	-	-	No major AEs
Peng et al. (16)	RFA±PEI	17	A, 52.9	3.1 (2.0–6.5)	-	14.5^*^	-	-	88	80	72^‡^	-	23.5% hematuria
			B, 47.1										
Kariyama et al. (11)	RFA	50	-	1.1 (0.5–4.0)	12	12	12	-	-	-	-	-	No major AEs
Fujimori et al. (17)	RFA+TACE	20	A, 65	2.2 ± 0.2^§^ (0.8–5.0)	6.3	-	13.5	13,5	94.4	-	86.6	67.5	10 % major AEs
			B, 35										
Hyun et al. (20)	TACE+RFA	14	A, 78.6	–( ≤ 2)	0	-	12.5	12.5	100	-	80.8	80.8	No major AEs
			B, 21.4										
Lee et al. (12)	RFA	43	A, 95.3	2.0 ± 0.9^§^	2.4	2.4	2.4	-	97.1	94	80.7	-	7% ≥G3 AEs
			B, 4.7										
	TACE	31	A, 90.3	2.0 ± 1.2^§^	18.6	32.9	38.5	-	89	80.8	62	-	0% ≥G3 AEs
			B, 9.7										
Shibata et al. (15)	PEI±TACE	25	A, 60	2.4 (1.4–5)	28^*^	-	-	-	70.6	60.2	48.1	16.0	No major AEs
			B, 36										
			C, 4										
Dou et al. (10)	MWA	20	A, 85	2.4 ± 0.9^§^ (1.1–4.8)	16.7^*^	-	-	-	-	-	-	-	No major AEs
			B, 15										
Kim et al. (18)	TACE	40	A, 72.5	2.5 (1.9–3.2)	51	-	64	-	92	79	65	56	Not reported
			B, 27.5										
Terayama et al. (19)	TACE	13	A, 53.8	2.5 (0.5–4)	75^†^	-	-	-	89	-	74	-	Not reported
			B, 23.1										
			C, 23.1										
Present study	RT	70	A, 90	2.0 (1.0–8.2)	6.1	15.0	15.0	19.4	85.6	73.6	68.8	51.3	5.7% ≥ G3 AEs
			B, 10										

*n, number; CPC, child-pugh classification; LP, local progression; y, year; MWA, microwave ablation; n, number of patients; ECOG PS, eastern cooperative oncology group performance status; HBV, hepatitis B virus; AFP, α-fetoprotein; Tx, treatment; TACE, transarterial chemoembolization; RFA, radiofrequency ablation; PEI; percutaneous ethanol injection; SR, surgical resection; SAT, systemic anticancer therapy; RT, radiotherapy; 3DCRT, 3-dimensional conformal RT; IMRT, intensity-modulated RT; SBRT, stereotactic body RT; PBT, proton beam RT; _TL_V30Gy, relative volume of the total liver receiving ≥30 Gy_3_; _RL_V30Gy, relative volume of the remaining liver receiving ≥30 Gy_3_; _RL_AV30Gy, absolute volume of the remaining liver receiving <30 Gy_3_; _GI_V45Gy and _GI_V50Gy, absolute volume of gastrointestinal (GI) organs receiving ≥45 Gy_10_ and ≥50 Gy_10_, respectively; _GI_Dmax, delivered radiation dose to GI organs. CI, confidence interval*.

* *Overall rate*.

† *6 months*.

‡ *4-year*.

§ *Mean ± standard deviation*.

RT using 3DCRT, IMRT, SBRT, and PBT has recently been attempted for HCC patients in various clinical situations, such as those with recurrent disease after other local treatments and difficult-to-treat disease with other local treatments, and its local tumor control effects and safety have been reported in previous studies (21–30). Several retrospective studies comparing SBRT with RFA showed that SBRT showed a 2-year FFLP rate of 80.6–83.8% and safe toxicity profiles (i.e., ≥grade 3 AE rate of <5%) (24, 26). In addition, a recent randomized controlled trial comparing PBT with RFA for recurrent HCC patients showed that PBT was not inferior to RFA in the 2-year FFLP rate (94.8 vs. 83.9%, *p* = 0.123) and was safer in toxicity profiles (≥grade 3 AE rate: 0 vs. 16.1%, *p* = 0.001) (30). However, to date, there has been no study of RT for HCC in the caudate lobe evaluating its effectiveness and safety until now. The present study was the first to evaluate the clinical outcomes of RT for HCC in the caudate lobe using a multi-institutional database due to the rarity of HCC in the caudate lobe. In the present study, RT for HCC in the caudate lobe showed promising clinical outcomes in terms of the 5-year FFLP and OS rates (80.6 and 51.3%, respectively), and ≥grade 3 AE rate (5.7%). Although direct comparisons of the present study with previous studies using ablative treatments and TACE were not possible, the FFLP and OS rates of RT for HCC in the caudate lobe were at the upper ends of a wide range according to the results of previous studies, and the ≥grade 3 AE rates were comparable (10–20) (Table 6).

In the present study, the radiation dose was significantly associated with the FFLP rate (HR, 0.57 per 10 Gy_10_ increase, *p* = 0.001), and the status of FFLP was significantly associated with OS (HR, 2.694, *p* = 0.014) (Table 4). These findings suggest that local tumor control increases with increasing radiation dose and that increased local tumor control may subsequently increase OS. However, not surprisingly, the radiation dose was negatively correlated with the PTV (*r*= −0.574, *p* < 0.001), and the PTV was positively correlated with the irradiated volume of the liver (_TL_V30Gy and _RL_V30Gy) and GI organs (_GI_V45Gy and _GI_Dmax.). These findings suggest that as the tumor volume increases, the irradiated volumes of the surrounding normal tissues, such as the liver and GI organs, inevitably increase. In the present study, no local progression was observed in patients receiving >90 Gy_10_, and the incidence of ≥grade 2 hepatic and GI AEs was significantly lower in patients with _TL_V30Gy of ≤ 15% and _RL_V30Gy of ≤ 10% and _GI_V45Gy of ≤ 1.0 cm^3^ and _GI_Dmax of ≤ 45.0 Gy_10_, respectively (*p* < 0.05 each) (Table 5). Although further large-scale studies are needed to identify the optimal radiation dose for local tumor control and dose-volumetric parameters for the liver and GI organs to avoid severe AEs, the present study suggests that a radiation dose >90 Gy_10_ be considered to achieve sufficient local tumor control while maintaining _TL_V30Gy of ≤ 15% and _RL_V30Gy of ≤ 10% and _GI_V45Gy of ≤ 1.0 cm^3^ and _GI_Dmax of ≤ 45.0 Gy_10_ for avoiding hepatic and GI AEs, respectively.

The present study has several inherent limitations. First, this study was a retrospective analysis that included a heterogeneous population (those with recurrent disease and/or difficult-to-treat disease after/with other local treatment modalities, various RT techniques and dose-fractionation regimens, and a history of various pre- and post-treatment modalities); thus, potential selection bias was not thoroughly assessed. However, because of the rarity of HCC in the caudate lobe, the present study included a relatively large number of patients treated with RT using a multi-institutional database. In addition, although direct comparisons of RT with other treatments modalities were not performed, in present study, RT showed comparable FFLP and OS rates with those of previous studies on other non-surgical treatments (10–20). Second, in the present study, RT for HCC in the caudate lobe showed a favorable safety profile, with a ≥grade 3 AE rate of 5.7%, but retrospective studies are likely to underestimate AEs due to recall bias, the incompleteness of medical records, etc. Similar to the present study, several retrospective studies of RT using 3DCRT, IMRT, SBRT, and PBT in HCC patients have shown favorable safety profiles (21–30). In addition, the ≥grade 3 AE rate in the present study was comparable with those of previous studies on other non-surgical treatments (10–20). However, because the population of the present study was relatively small and comprised heterogeneous populations and detailed treatments, further large-scale studies are warranted.

In conclusion, the present study demonstrated that RT for HCC in the caudate lobe resulted in promising clinical outcomes in terms of the 5-year FFLP and OS rates (80.6 and 51.3%, respectively), which are comparable to those observed in other local treatments for HCC in the caudate lobe, and RT was tolerable and safe, consistent with its known profile. These findings suggest that RT may be a promising treatment option for HCC in the caudate lobe.

## Data Availability Statement

The raw data supporting the conclusions of this article will be made available by the authors, without undue reservation.

## Ethics Statement

The studies involving human participants were reviewed and approved by National Cancer Center (20190300), Asan Medical Center (20200428), and National Taiwan University Hospital (202002045RINA). Written informed consent for participation was not required for this study in accordance with the national legislation and the institutional requirements.

## Author Contributions

TK, SY, and JC: conceptualization and supervision. TK, SY, JC, and SL: data collection. TK and SL: formal analysis. TK, SY, JC, BK, J-hP, JJ, C-LT, YC, and J-WP: investigation. SL and TK: writing the original draft. All authors reviewed and approved the final version of the manuscript.

## Conflict of Interest

The authors declare that the research was conducted in the absence of any commercial or financial relationships that could be construed as a potential conflict of interest.
