# Supplementary material for: Multi-Institutional Retrospective Study of Radiotherapy for Hepatocellular Carcinoma in the Caudate Lobe
Source: Front Oncol. 2021 Feb 26;11:646473. doi: 10.3389/fonc.2021.646473 (PMC7952981; doi:10.3389/fonc.2021.646473)
Supplement: Supplementary file 1 [file Data_Sheet_1.pdf]

Supplementary Material

1 Supplementary Table

Supplementary Table 1. Subsequent treatment modalities for disease progression

Details of subsequent Tx		Distribution, n (%)
Post-Tx to target lesion(s)	No	64 (91.4)
	Yes	6 (8.6)
	TACE	5 (7.1)
	PEI	1 (1.4)
Post-Tx to non-target lesion(s)	No	16 (22.9)
	Yes	52 (74.2)
	TACE ± RFA/PEI/RT	28 (40.0)
	SAT* ± TACE/RFA/RT	15 (21.4)
	LT ± TACE/RFA/RT	4 (5.7)
	RFA	3 (4.3)
	SR ± TACE/RFA	2 (2.9)

Abbreviations: n, number of patients; Tx, treatment; TACE, transarterial chemoembolization; RFA, radiofrequency ablation; PEI, percutaneous ethanol injection; SR, surgical resection; RT, radiotherapy; SAT; systemic anticancer therapy; LT, liver transplantation

*Sorafenib (n=13), thalidomide and tegafur/uracil (n=2), axitinib (n=1), and nivolumab (n=1).

2 Supplementary Figures

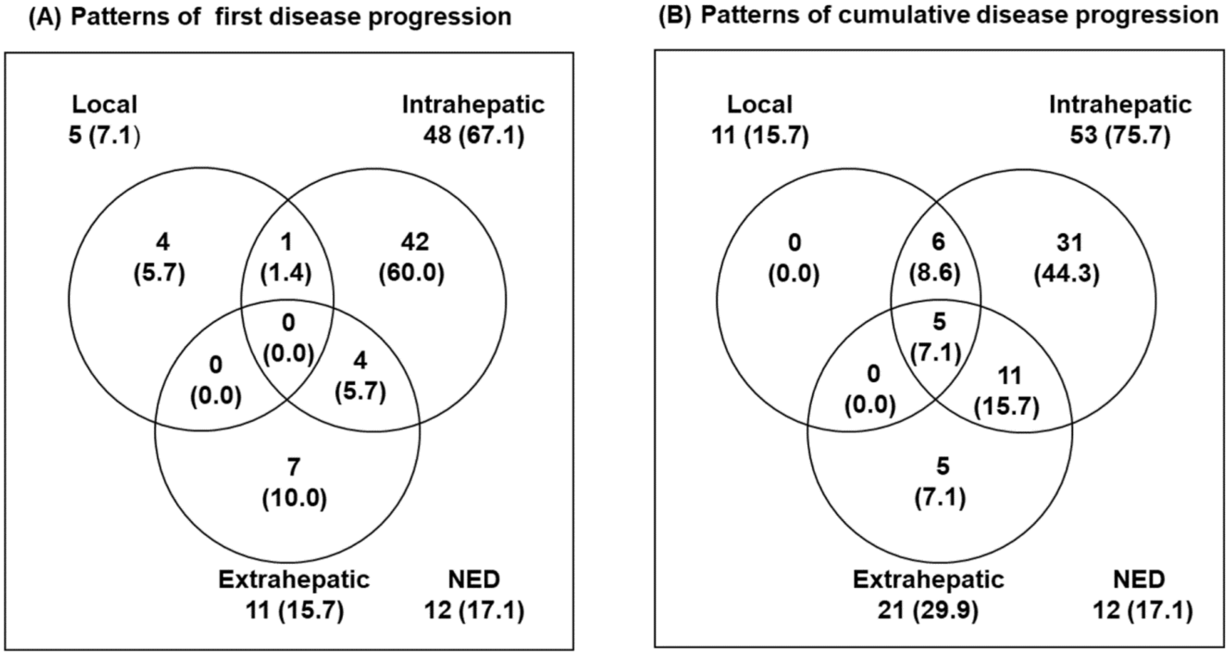

Supplementary Figure 1. The patterns of first (A) and cumulative (B) disease progression after radiotherapy for hepatocellular carcinoma in the caudate lobe

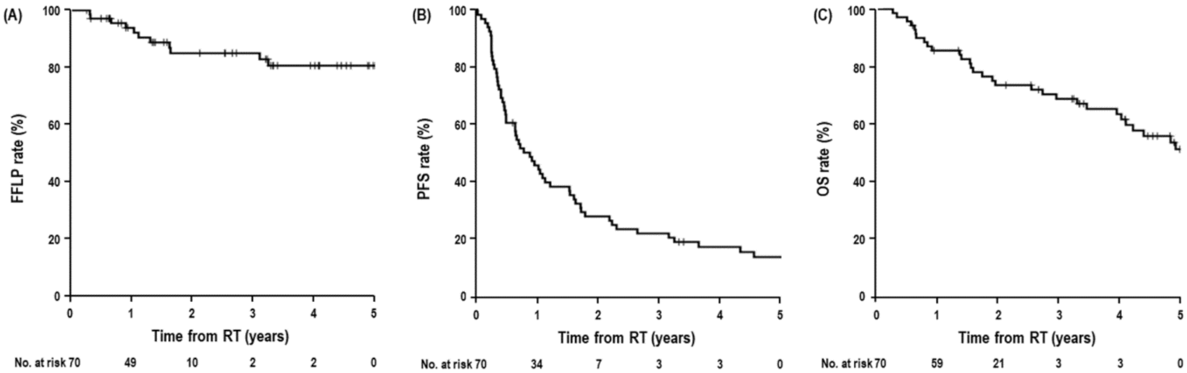

Supplementary Figure 2. Freedom from Local progression (FFLP) (A), progression-free survival (PFS) (B), and overall survival (OS) (C) curves in patients with hepatocellular carcinoma in the caudate lobe treated with radiotherapy (RT).
